# The Effects of Coconut Skim Milk and Coco-Dairy Milk Blend on the Nutritional Status of Schoolchildren

**DOI:** 10.1155/2022/6793866

**Published:** 2022-09-19

**Authors:** Imelda A. Agdeppa, Jezreel Ann T. Zamora

**Affiliations:** Food and Nutrition Research Institute, Department of Science and Technology Compound, General Santos Avenue, 1632 Lower Bicutan, Taguig, Philippines

## Abstract

Milk feeding can be an effective response to the high prevalence of child undernutrition as it provides significant amounts of nutrients. This study investigated and compared the effects of coconut skim milk (CocoM) and coco-dairy milk blend (CDMB) to cow's milk (CM) on improving the nutritional status of Filipino schoolchildren. The study followed a randomized, double-blind, controlled, parallel-group design. A total of 444 underweight/stunted schoolchildren aged 6.0–8.4 years old enrolled in Guadalupe Elementary School in Cebu City, Philippines, have participated. The participants were randomly allocated into three groups, that is, CocoM, CDMB, and CM, in which the milk products were packed in 200 ml color and number-coded bottles given for 95 days. The bottles were similar in form and shape, and the only differentiating factors were the code and color. Weight and height were measured using standard techniques. Nutritional indices such as weight-for-age *z*-score (WAZ), height-for-age *z*-score (HAZ), and BMI-for-age *z*-score (BAZ) were measured using the WHO Child Growth Standards (CGS), and the acceptability test was measured using the 5-point facial hedonic scale. The study found that the mean WAZ and BAZ had significantly increased from baseline to endpoint across all groups. Moreover, the prevalence of underweight has also significantly declined from baseline to endpoint in all groups, and the prevalence of stunting significantly declined from baseline to endpoint in the CocoM group only. Lastly, the three milk products were rated as generally acceptable. Overall, the findings indicate that coconut milk consumption could be beneficial for improving a child's WAZ and BAZ, as well as improving the nutritional status of underweight and stunted schoolchildren. CocoM and CDMB were found to be equally beneficial to child nutrition as cow's milk. Hence, the present study suggests that CocoM and CDMB could be also provided in school-based feeding programs with the aim of targeting child undernutrition.

## 1. Introduction

Undernutrition is associated with 45% of child deaths around the world [1]. In the Philippines, the 2013 National Nutrition Survey data revealed that 29.1% were underweight, and 29.9% were stunted among 5–10-year-old Filipino schoolchildren [2]. Children are most vulnerable to undernutrition due to their insufficient dietary intake, poor food accessibility, and inequitable distribution of food within households [3].

One way of targeting child undernutrition is through school supplementary feeding programs, which are intended to provide food for undernourished children and help improve their health. School feeding programs are geared towards alleviating hunger and preventing malnutrition [4, 5].

In the Philippines, the Department of Education (DepEd) implemented the School-Based Feeding Program (SBFP) through the School Health Division-Bureau of Learner Support Services [6]. It specifically aims to improve a child's nutritional status by at least 70% towards the end of a 120-day feeding period and aims to improve the child's health and nutritional behavior [7].

Under the Department of Social Welfare and Development (DSWD) Administrative Order No. 04 series of 2016 as amended by DSWD Memorandum Circular No. 3 series of 2019 and DSWD Memorandum Circular No. 12 series of 2020, the milk supplementation of children will be in addition to their regular meals (hot meals or alternative meals) in the Philippines [8]. This Milk Feeding Program also serves as support to local dairy industry, which will contribute to the sustainable economic activities of the local farmers, as stated by the Philippine Carabao Center. This is supported by the Republic Act 7884, or the National Dairy Development Act of 1995, which requires that government nutrition programs that utilize milk shall be supplied by local milk producers and shall be in correspondence with the National Dairy Authority (NDA) [9]. DepEd also decided to start milk feeding in April 2022 as part of their School-Based Feeding Program (SBFP) [10]. With regards to these, the impact of daily milk supplementation has been proven as previous studies found an increase in height and weight [11] and an improvement in the micronutrient intakes among children [12, 13].

Aside from dairy milk drawn from cows, milk could also be derived from plant sources such as coconuts. Additionally, coconut milk, like other plant-based milks, does not contain lactose, making it suitable for people with lactose intolerance [14]. Coconuts are considered the main export agricultural commodity in the Philippines [15]. As reported in a previous study, wherein the NDA conducted a supplementary feeding program in Cebu City, Philippines, the provision of coconut milk increased the height of children [16]. The increase in height may be attributed to the health-beneficial qualities of coconut milk. First, unlike other plant-based alternatives to conventional milk, coconuts are higher in medium-chain triglycerides (MCT), whereas most fat sources majorly consist of long-chain triglycerides (LCT) [17]. Furthermore, the fat that is present in coconuts is less likely to clog arteries because the body does not store coconut fats, which makes coconut milk a healthy alternative to cow's milk when it comes to preserving heart health [18]. Aside from this, coconut milk's soluble and insoluble fiber content and antioxidant properties increase its nutritional value [19]. It is also rich in micronutrients such as vitamin C, vitamin B, iron, and phosphorus [20].

The aforementioned qualities of coconut milk altogether confirm that it is indeed a functional food. Yet, no previous research focusing on the effects of coconut milk on the nutritional status of schoolchildren has been recorded in the Philippines. In the context of research, the effects of plant-based milk to gain supposed health effects aside from the usual cow's milk provided during milk feedings in the Philippines have not yet been explored, have not been supported by adequate scientific evidence, and have no proved benefits for children. Thus, cow's milk was regarded in the present study as the control product in order to measure if coconut milk-based drinks could also positively impact child nutrition.

This study aimed to investigate and compare the effects of consuming coconut skim milk (CocoM), coco-dairy milk blend (CDMB), and cow's milk on the nutritional status of Filipino schoolchildren over a 95-day feeding period.

Findings of the study could be used as evidence-based information towards the development of nutrition interventions for undernourished children. The present study also supports the Republic Act No. 11037 on Child Nutrition, which is an Act Institutionalizing a National Feeding Program for Undernourished Children in Public Day Care, Kindergarten, and Elementary Schools to Combat Hunger and Undernutrition among Filipino Children [21]. Aside from these, the study also aims to promote an increase in the demand for coconuts, thereby also helping local farmers increase their income.

## 2. Materials and Methods

### 2.1. Sample Population and Study Design

The study followed a randomized, double-blind, controlled, parallel-group design and was performed at Guadalupe Elementary School in Cebu City, Philippines. This school was referred by the Department of Education (DepEd) Region VII since it has a high number of underweight children based on their records. The research was carried out between August and November of 2015, and the study included 444 underweight and stunted schoolchildren aged 6.0 to 8.4 years old.

### 2.2. Sample Size

The sample size was computed using the results of a previous study on supplementary feeding that investigated the effects of the multinutrient fortified juice drink on the iron, zinc, and anthropometric indices of children [22]. Using the G Power software [23], sample size was calculated with an effect size of 0.15, (alpha) error of 0.10, and (beta) power of 90%. The sample size is computed at 117 per group, or a total of 351 children, based on the primary outcomes of mean weight increase at endpoint. To allow for a drop-out rate of 22%, the sample size was set at 150 per group, or a total of 450.

### 2.3. Statistical Analysis

Stata version 15 was utilized in all analysis and data management. An analysis of Covariance (ANCOVA) was used to test the difference of anthropometric indices between the two treatment groups (CocoM and CDMB) and the control group (CM). Repeated measure analysis of variance (RM-ANOVA) was used to test the mean difference of each anthropometric index within time periods. Pearson's *χ*^2^ test was used to test the association between nutritional indices and treatment group, while McNemar test was used to test the significant change of nutritional indices from baseline to endpoint. All tests of hypothesis were used with a 95% level of significance. The analysis only included children who had a complete data set.

### 2.4. Screening of Participants

Children who (1) were underweight and/or stunted, (2) had no physical abnormality, (3) were not suffering from diarrhea, respiratory infections, or fever at the time of interview or in the previous two weeks prior to the interview, (4) had not participated in any nutrition program for the previous four months, and (5) had an informed consent form signed by parent or guardian for children ages 6.0 to 6.9 years and an informed assent form signed by children ages 7.0 to 8.4 years were included in the study. All children who did not comply or met the inclusion criteria were excluded from the study.

### 2.5. Study Products and Intervention

Three forms of milk were used in the study: coconut skim milk, coco-dairy milk blend made up of 80% cow's milk and 20% coconut skim milk, and the control product, which is cow's milk. The CDMB is composed of 80% cow's milk and 20% coconut skim milk, so that higher carbohydrate content can be observed. The milk drinks were in liquid form and were given in individual 200 mL unbranded bottles. The bottles used in the study were similar in form and shape, and the only differentiating factors were the code and color. Since the interest of the study is on stunting and underweight, which focuses more on macronutrient intake, the micronutrient content was not analyzed, which was also due to financial constraint. The following were the methods used for analyzing the macronutrient content of the three types of milk: total carbohydrate through computation by difference from the results of moisture, ash, protein, and fat; protein through automated Kjeldahl; fat through acid hydrolysis (Soxhlet); moisture and ash through the gravimetric method.  Table 1 shows the nutrient content of each milk drink from the NDA.

Before the start of intervention, the random assignment of treatments by group was predrawn. The study participants and the research team were both blinded to treatment group assignment. All screened underweight and stunted children were randomly allocated into three groups using a simple random technique. A staff at NDA randomly assigned the children into groups with respective numbered labels and color codes for the different types of milk. The codes and labels were kept in a sealed envelope, and the packaging of individual milk products was supervised. The labels were decoded after the statistical analysis was done by the research team.

For the conduct of intervention, each of the children was given color-coded identification cards (IDs) corresponding to their group code as per study design. The feeding was also done by group per room by code. The research assistant (RA) directly gave one bottle of 200 ml milk drink to the children according to the color of their IDs matched with the color of the milk bottles from Mondays to Fridays during recess (9:30 h to 10:00 h) under a supervised regimen for a total of 95 days. The milk intervention was intended for 120 days. However, due to numerous holidays and suspension of classes, milk feeding days were reduced to 95 days.

The different types of milk were produced and delivered directly to the study site every day. The children were instructed to consume the milk together with a 30 g pack of plain, nonfortified biscuit. The biscuit is given to avoid the incidence of gastric irritation. It is intended to maintain zero leftovers all throughout the study. However, in cases where there was leftover, actual consumption was measured by deducting the volume of leftover from the actual volume of 200 mL. Empty bottles were collected and inspected after each feeding session by the RAs. Attendance and milk consumption were monitored and recorded on a prescribed case report form (CRF). No adverse events, however, were encountered during the study.

### 2.6. Deworming

Deworming was conducted prior to feeding. The final lists of participants were cross-checked with the school's master list of children who were given deworming medicine during the National Deworming Day on July 30, 2015. All child participants not found in the National Deworming Day list were given 400 mg of Albendazole and were administered by the DepEd physician and nurses.

### 2.7. Measurements

Parents or caregivers of child participants were interviewed face-to-face using a pretested structured questionnaire. The interview included the general profile of the child (age and sex), socioeconomic demographic, and sanitation data.

The weight and height were measured at baseline (screening), midline (47 days), and endpoint (95 days). Weight of participants was measured using a calibrated SECA 874 digital weighing scale (Hamburg, Germany) and recorded to the nearest 0.1 kg. Participants wore light clothing and wore no shoes. Height measurement was conducted through a microtoise (Depose, France) placed flat against a wall with the children standing straight and barefoot and was recorded to the nearest 0.1 cm. Each anthropometric measurement was repeated twice, and the average was used for analysis. A third measurement was obtained if the difference between the first two measurements was greater than 0.5 cm or 0.3 kg. Hence, the average of the two nearest measurements was used for analysis. Body mass index (BMI) was calculated by dividing weight in kilograms by height in meters squared (kg/m^2^). Reference standards are all based on BMI cutoff points [24], which are sex- and age-specific. BMI was also calculated to measure changes in mean BMI among children.

In terms of the nutritional indices, weight-for-age (WAZ), height-for-age (HAZ), and BMI-for-age *z*-scores (BAZ) were used in the study (WHO). Underweight was defined as WAZ below two standard deviations of the WHO Child Growth Standards (CGS) median, stunting was defined as HAZ below two standard deviations of the WHO CGS median, and wasting was defined as BAZ below two standard deviations of the WHO CGS median [25].

### 2.8. Dietary Assessment

Food intake was collected at baseline and endpoint through a face-to-face interview with a parent or guardian of the child using a 24-hour food recall questionnaire administered on two nonconsecutive days. The previous day's food intake (24 hrs) of the child on a weekday was asked. This was recorded on a validated dietary assessment form used by the Department of Science and Technology–Food and Nutrition Research Institute (DOST-FNRI) for the National Nutrition Survey and other projects. The quantity of intake was estimated using standard household measures that were provided by each RA during the interview. Food intake was translated to nutrient intake through the Individual Dietary Evaluation System (IDES) developed by DOST-FNRI. The mean nutrient intake was computed in the three groups.

### 2.9. Acceptability of Milk Products

The RAs administered a 5-Point Facial Hedonic Scale questionnaire based on appearance, odor, taste, and overall liking parameters to all child participants by the RAs after a week of consumption and every month thereafter until the end of the 95-day feeding period with the following rating: 5 = Very Much Like, 4 = Moderately Like, 3 = Neither Like nor Dislike, 2 = Dislike Moderately, and 1 = Dislike Very Much.

## 3. Results

### 3.1. Profile of Child Participants

#### 3.1.1. Age and Gender

A total of 3, 356 children from grade levels 1–3 aged 6.0 to 8.4 years old in the study site were invited to participate in the study to undergo anthropometric measurements. After considering the inclusion and exclusion criteria, only 444 children were qualified to participate in the study and were randomly allocated into three groups. However, only 317 (70.44%) children completed the data set from baseline to endpoint (see Table 2for Distribution of Drop-Outs). The distribution of children by age and sex at baseline according to age group showed that the mean age was 7.23 years old and that about 52.7% were females, and 47.3% were males (Table 3).

### 3.2. Socioeconomic and Demographic Profile

#### 3.2.1. Household Profile

Half of the child participants' family structure was nuclear (59.9%) with an average family size of 6 (see Table 4). For parents' educational attainment, the majority of mothers (38.8%) and fathers (28.4%) were high school graduates. Some were degree holders (12.0% of mothers and 13.6% of fathers), while others were college undergraduates (13.6% of both mothers and fathers). Moreover, most of the families interviewed dwell in single houses (88.6%), half of them own the house they live in (53.3%), about a quarter were either living with their relatives or considered caretakers of the house (25.9%), more than a quarter have their own lot (28.4%), 23.75% were renting, and a few were informal settlers (17.0%) (see Table 4). Most households have refrigerators (37.9%). It is one of the two main food storage methods used by families, aside from simply placing the food on a table with a cover (26.5%). Their drinking water source was mostly purified water (75.1%), and nearly all of the households disposed of their waste through their local garbage collection system (85.8%) (see Table 4).

#### 3.2.2. Occupation, Income, and Food Expenditure

The top three major occupations of the household head were service-related (40.7%), production-related (18.0%), and transportation-related (16.7%) jobs. A few household heads are involved in business or self-employed, clerical related, agriculture-related, professional, and technical related jobs. Moreover, most of the households had only one earning family member (67.5%), and more than a quarter had two earning members (28.4%). The mean estimated family monthly income was approximately PHP 9, 343.22, and the mean estimated daily food expenditure was approximately PHP 239.00 (see Table 4).

### 3.3. Anthropometric Indices Based on *Z*-Scores (WHO-CGS)

The results of the mean WAZ, HAZ, and BAZ of children by group and time period are shown in Table 5. At baseline, the CocoM and CDMB groups had higher mean WAZ than the cow's milk group and continued to be higher in midline and end line. The mean HAZ and BAZ do not differ across groups at all time points. Further, mean WAZ significantly increased from baseline to end line for each group. Mean HAZ significantly decreased from baseline to end line for the CDMB group only. Mean BAZ significantly increased from baseline to end line for each group.

### 3.4. Prevalence of Underweight, Stunting, and Wasting

The results reveal that the prevalence of underweight has significantly declined from baseline to endpoint in all groups, where the percentage decrease was similar in all three groups across time periods. Furthermore, the prevalence of stunting, underweight, and wasting does not significant differ across groups at end of the intervention (see Table 6).

### 3.5. Acceptability of Milk Products

**Figure 1:**
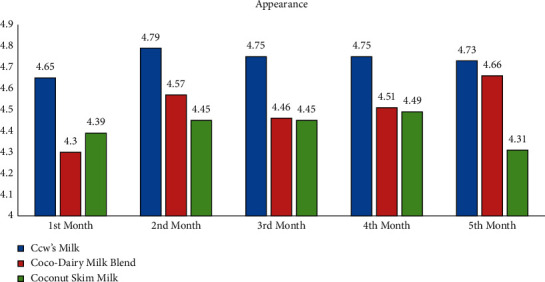
Average acceptability scores of milk products according to appearance by months.

**Figure 2:**
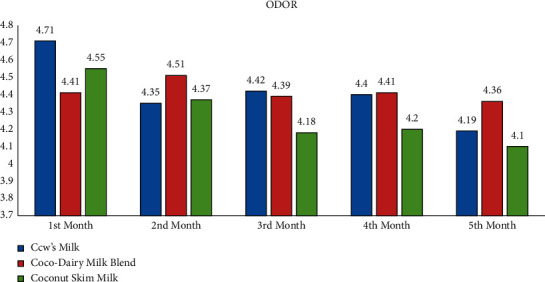
Average acceptability scores of milk products according to odor by months.

**Figure 3:**
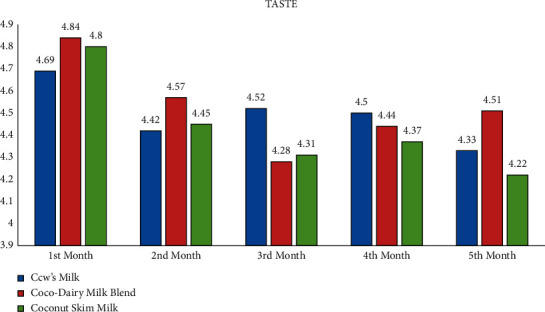
Average acceptability scores of milk products according to taste by months.

**Figure 4:**
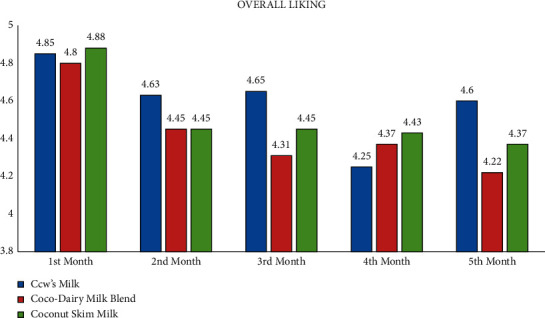
Average acceptability scores of milk products according to overall liking by months.

The study revealed the average scores of the acceptability test using the 5-point Facial Hedonic Scale based on appearance, odor, taste, and overall liking parameters. It is revealed that, for all mentioned parameters, CDMB, CocoM, and CM were evaluated by child participants as “Like Very Much” and “Like Moderately” for the duration of five months. Thus, CocoM and CDMB were deemed generally acceptable by child participants as much as CM (see Appendix: Figures 1–4).

## 4. Discussion

Using a representative sample of school-aged children in the Philippines, this study investigated and compared the effects of coconut skim milk (CocoM) and coco-dairy milk blend (CDMB) on improving the nutritional status of Filipino schoolchildren The CocoM and CDMB groups had higher mean WAZ than the cow's milk group at baseline, and this trend remained through midline and end line. At all time points, the mean HAZ and BAZ do not differ across groups. Furthermore, mean WAZ rose considerably from baseline to end line for each group. The CDMB group's mean HAZ fell considerably from baseline to end line. Each group's mean BAZ grew considerably from baseline to end line.

The results of the present study had the same findings as a previously conducted randomized controlled trial in Kenya, wherein significant changes for WAZ, but not for HAZ, were found among children [26]. Lien et al. found a significant change in the HAZ, but not in the WAZ, among Vietnamese school-aged children [27]. Yet, these studies were country-specific, differed in their type and measurement of milk, and focused on different age groups, ranging from under-five children to children aged 6 to 14 years, which reduced their comparability and presented the need for country-specific data. Underweight prevalence decreased significantly from baseline to endpoint in all groups, while stunting prevalence decreased significantly from baseline to endpoint in the CocoM group only.

Improvements in the nutritional status of school-aged children were observed, which may be due to considering the abundance of nutrients and minerals in coconut milk, including iron, calcium, potassium, magnesium, and zinc [28]. Coconut protein is also found to be rich in amino acids such as lysine, methionine, and tryptophan, which are vital for child growth and nutrition [29, 30]. Moreover, previous research stated that coconut milk can play a vital role in balancing the nutritional deficiency of one's diet [31] although the correlation between nutrient intakes through coconut milk and nutritional status improvement was not explored in the present study.

Even though stunting is considered irreversible, improved nutritional intake is still important, and further damage to nutritional status needs to be prevented [32], especially as stunted children have diminished absorption of nutrients and reduced appetite [33]. In addition, girls and boys of this age group have the potential to experience catch-up growth, that is, growth that will bring them to a normal range of height for age [34]. A previous study also stated that there is potential for remediation of early growth deficits through interventions [35]. Regarding the acceptability, it was revealed that all study products were deemed as generally acceptable by child participants. Considering all parameters, all study products were evaluated by child participants as “Like Very Much” and “Like Moderately.” These findings were consistent with a previous study in Pakistan, wherein a sensory and nutritional evaluation was conducted for coconut-natural milk blend and cow's milk, and the coconut-natural milk blend got a higher rate with a mean score of 8.6 out of 9.0 compared to cow's milk with a mean score of 7.0 out of 9.0 [36]. The aforementioned study also stated that although the consumers were not familiar with the taste of coconut-natural milk blend, they still preferred it over cow's milk due to the mild taste of coconut flavor [36].

Hence, the present study found that CocoM and CDMB had no difference with CM. Therefore, CDMB and CocoM could be used for the supplementary milk feeding of schoolchildren for the purpose of eliminating the increasing prevalence of undernutrition. Moreover, the study's findings support the National Feeding Law, or Republic Act 11037, which states that fresh milk products should be included in fortified meals and cycle menus for children suffering from undernutrition in public day care, kindergarten, and elementary schools in order to enhance the nutrition of schoolchildren [7].

## 5. Conclusion

This study adds to the body of knowledge because it is the first to quantify how coconut milk-based beverages affect school-aged children's nutritional status. Overall, the results suggest that consuming coconut milk may help children achieve better weight-for-age and BMI-for-age z-scores and less frequently experience underweight and stunting. It has been discovered that coconut skim milk (CocoM) and coco-dairy milk blend (CDMB) are just as healthy for children's nutrition as cow's milk. However, only in the CocoM group did the prevalence of stunting considerably decrease from baseline to endpoint. In order to combat child malnutrition, the current study recommends that beverages made with coconut milk could also be offered in school-based feeding programs. We suggest that more studies comparing the different types of plant-based milk with cow's milk are needed to be able to draw other comparisons. More research is required to further investigate the causality between coconut milk consumption and its impact on a child's nutritional status and dietary intake.

## 6. Limitations of the Study

The baseline WAZ, HAZ, and BAZ for the control group are significantly lower than those for the two treatment groups, which raises the possibility of bias. This could alter the findings, which show that the CDBM and CocoM groups' anthropometrics are improved relative to the CM group.

## Figures and Tables

**Table 1:** Comparative nutrient analyses of milk products.

Parameters	Coconut skim milk^*∗*^	Cow's milk^*∗*^	Coco-dairy milk blend^*∗*^
Energy (kcal/100 g)	41.4	64.3	61.9
Carbohydrate (g/100 g)	4.0	4.9	3.8
Protein (g/100 g)	4.1	3.3	3.8
Fat (g/100 g)	1.0	3.5	3.5

**Table 2:** Distribution of dropouts by group.

	Cow's milk		Coco-dairy milk		Coconut skim milk	
	*n* = 148		*n* = 148		*n* = 148	
	*n*	%	*n*	%	*n*	%
Relocation/transferred to other school	3	2	6	4	8	5.4
Participation withdrawal by parents/guardians	4	2.7	9	6.1	8	5.4
No endpoint data (anthropometry)/left for vacations in other places	19	2.8	38	25.7	32	23
Total drop out	26	17.6	53	35.8	48	32.4
Total endpoint count	122	82.4	95	64.2	100	67.6

**Table 3:** Profile of child participants.

	All		Control group		Intervention groups			
			Cow's milk		Coconut skim milk		Coco-dairy milk blend	
			*n* = 122		*n* = 100		*n* = 95	
	*n*	%	*n*	%	*n*	%	*n*	%
Characteristics								
Age, mean	7.23		7.2		7.2		7.3	

Age								
6	68	21.5	27	22.1	20	20	21	22.1
7	127	40.1	46	37.7	47	47	34	35.8
8	103	32.5	41	33.6	27	27	35	36.8
9	19	6	8	6.6	6	6	5	5.3

Sex								
Male	167	52.7	64	52.5	49	49	54	56.8
Female	150	47.3	58	47.5	51	51	41	43.2

**Table 4:** Percentage distribution of socio-economic and demographic profile of study participants.

Variables	All		Control group		Intervention group			
			Cow's milk		Coconut skim milk		Coco-dairy milk blend	
	*n* = 317		*n* = 122		*n* = 100		*n* = 95	
	*n*	%	*n*	%	*n*	%	*n*	%
Family type								
Nuclear	190	59.9	70	57.4	61	61	59	62.1
Extended	127	40.1	52	42.5	39	39	36	37.9

Household size								
1–10 members	298	94	115	94.3	94	94	89	93.7
10–20 members	19	5.99	7	5.74	6	6	6	6.3

Mother's educational attainment								
No education	3	0.95	1	0.82	2	2	0	0
Some elementary	13	4.1	2	1.64	6	6	5	5.3
Elementary graduate	27	8.52	8	6.56	10	10	9	9.5
Some high school	40	12.6	14	11.5	15	15	11	11.6
High school graduate	123	38.8	56	45.9	36	36	31	32.6
Some vocational	17	5.36	6	4.92	8	8	3	3.2
Vocational graduate	7	2.21	4	3.28	0	0	3	3.2
Some college	43	13.6	13	10.7	14	14	16	16.8
College graduate	38	12	16	13.1	8	8	14	14.7
Others	0	0	0	0	0	0	0	0
Not sure	6	1.89	2	1.64	1	1	3	3.2

Father's educational attainment								
No education	1	0.32	1	0.82	0	0	0	0
Some elementary	21	6.62	7	5.74	10	10	4	4.2
Elementary graduate	28	8.83	9	7.38	8	8	11	11.6
Some high school	31	9.78	16	13.1	8	8	7	7.4
High school graduate	90	28.4	33	27.1	26	26	31	32.6
Some vocational	16	5.05	6	4.92	8	8	2	2.1
Vocational graduate	13	4.1	5	4.1	4	4	4	4.2
Some college	43	13.6	16	13.1	17	17	10	10.5
College graduate	43	13.6	15	12.3	10	10	18	19.0
Others	2	0.63	1	0.82	1	1	0	0.0
Not sure	29	9.15	13	10.7	8	8	8	8.4

Main occupation								
Agriculture (fishing and farming)	3	0.95	2	1.64	1	1	0	0.0
Production related worker	57	18	24	19.7	18	18	15	15.8
Service-related worker	129	40.7	52	42.6	33	33	44	46.3
Business/self-employed	24	7.57	10	8.2	6	6	8	8.4
Transportation related worker	53	16.7	15	12.3	23	23	15	15.8
Professional/technical	21	6.62	8	6.56	7	7	6	6.3
Clerical related worker	14	4.42	3	2.46	7	7	4	4.2
Others	12	3.79	7	5.74	3	3	2	2.1
Not sure	4	1.26	1	0.82	2	2	1	1.1

Number of earning family members								
0	1	0.32	0	0	1	1	0	0.0
1	214	67.5	81	66.4	64	64	69	72.6
2	90	28.4	35	28.7	31	31	24	25.3
3	6	1.89	3	2.46	1	1	2	2.1
4	3	0.95	2	1.64	1	1	0	0.0
5	1	0.32	1	0.82	0	0	0	0.0
6	2	0.63	0	0	2	2	0	0.0

Estimated monthly family income								
<Php 5000	55	17.4	25	20.5	17	17	13	13.7
Php 5000–10000	155	48.9	56	45.9	48	48	51	53.7
Php 10000–15000	43	13.6	13	10.7	16	16	14	14.7
>Php 15000	42	13.3	18	14.8	11	11	13	13.7
Not sure	22	6.94	10	8.2	8	8	4	4.2
Estimated monthly family income, mean			9451.36		8945.6		9632.7	

Food expenditure								
<250 per day	206	65	79	64.8	56	56	71	74.7
>250 per day	99	31.2	37	30.3	38	38	24	25.3
Not sure	15	4.73	6	4.92	9	9	0	0.0
Food expenditure, mean			237.2		254.09		225.25	

Type of dwelling unit								
Single house	281	88.6	107	87.7	93	93	81	85.3
Duplex	0	2.84	4	3.28	1	1	4	4.2
Apartment/condominium/tenement/BLISS	3	0.95	1	0.82	0	0	2	2.1
Commercial	1	0.32	1	0.82	0	0	0	0.0
Makeshift	15	4.73	4	3.28	4	4	7	7.4
Others, specify	7	2.21	4	3.28	2	2	1	1.1
Not sure	1	0.32	1	0.82	0	0	0	0.0

Status of house								
Own	168	53	67	54.9	52	52	49	51.6
Rent	56	17.7	19	15.6	17	17	20	21.1
Free (owned by parents/relatives, caretaker)	82	25.9	30	24.6	27	27	25	26.3
Squat (settle in public/private dwelling without permission)	11	3.47	6	4.92	4	4	1	1.1
Others	0	0	0	0	0	0	0	0

Food storage								
Refrigerator	120	37.9	42	34.4	35	35	43	45.3
Screened/enclosed cabinet	76	24	29	23.8	27	27	20	21.1
On table with cover	84	26.5	35	28.7	27	27	22	23.2
Others, specify	37	11.7	16	13.1	11	11	10	10.5

Source of water								
Waterworks	85	17.4	31	25.4	9	9	15	15.8
Deep well	23	7.26	6	4.92	13	13	4	4.2
Dug well/shallow well	0	0	0	0	0	0	0	0
Purified bottle water	238	75.1	84	68.9	78	78	76	80
Others	1	0.32	1	0.82	0	0	0	0

Garbage								
Garbage collection system	272	85.8	107	87.7	78	78	87	91.6
Open pit	11	3.47	4	3.28	4	4	3	3.2
Covered pit	2	0.63	2	1.64	0	0	0	0
Covered pit then burned	24	7.57	8	6.56	12	12	4	4.2
Open pit then burned	4	1.26	0	0	4	4	0	0
Throw anywhere	4	1.26	1	0.82	2	2	1	1.1
Others	0	0	0	0	0	0	0	0

Water disposal								
Water sealed	317	100	122	100	100	100	95	100
Sanitary pit privy	0	0	0	0	0	0	0	0
Bored hole latrine	0	0	0	0	0	0	0	0
Others	0	0	0	0	0	0	0	0

**Table 5:** Mean weight-for-age *z*-scores (WAZ), height-for-age *z*-scores (HAZ), and BMI-for-age *z*-scores (BAZ) of child participants by group and time periods.

	Control group	Intervention groups		*p* value^1^
	Cow's milk	Coconut skim milk	Coco-dairy milk blend	
	*n* = 122	*n* = 100	*n* = 95	
WAZ				
Baseline	−2.44 (0.62)	−2.19 (0.62)	−2.19 (0.64)	0.031^*∗*^
Midline	−2.28 (0.69)	−2.01 (0.68)	−2.02 (0.69)	0.029^*∗*^
End line	−2.33 (0.69)	−2.10 (0.69)	−2.09 (0.70)	0.039^*∗*^
*p* value^2^	<0.001^*∗*^	<0.001^*∗*^	<0.001^*∗*^	

HAZ				
Baseline	−2.37 (0.64)	−2.35 (0.65)	−2.22 (0.52)	0.342
Midline	−2.38 (0.64)	−2.35 (0.63)	−2.21 (0.52)	0.298
End line	−2.40 (0.65)	−2.37 (0.65)	−2.26 (0.53)	0.461
*p* value^2^	0.259	0.124	<0.001^*∗*^	

BAZ				
Baseline	−1.24 (0.87)	−0.90 (0.87)	−1.03 (0.92)	0.147
Midline	−1.00 (0.86)	−0.65 (0.83)	−0.81 (0.92)	0.077
End line	−1.07 (0.86)	−0.76 (0.85)	−0.87 (0.95)	0.083
*p* value^2^	<0.001^*∗*^	<0.001^*∗*^	<0.001^*∗*^	

^1^Analysis of covariance (ANCOVA) adjusted for age, sex, household size, household estimated monthly income, mother's educations, and father's education. ^2^Repeated measure analysis of variance (ANOVA); ^*∗*^significant at *α* = 0.05; ^a^cow's milk is significantly different (lower) than coco-dairy milk blend and coconut skim milk intervention groups. Values were mean (standard deviation).

**Table 6:** Prevalence of underweight, stunting, and wasting among child participants.

Nutritional indices	Control group		Intervention groups				*p* value^2^
	Cow's milk *n* = 122		Coco-dairy milk blend *n* = 95		Coconut skim milk *n* = 100		
	*n*	%	*n*	%	*n*	%	
Underweight							
Baseline	96	78.7	66	699.5	68	68	0.15
Endpoint	81	66.4	49	51.6	59	59	0.087
Change	−15	12.3	−17	17.9	−9	9	
*p* value^1^	0.001^*∗*^	0.000^*∗*^	0.049^*∗*^				

Stunting							
Baseline	93	76.2	74	77.9	83	83	0.452
Endpoint	92	75.4	69	72.6	74	74	0.897
Change	−1	0.8	−5	5.3	−9	9	
*p* value^1^	1	0.180	0.012^*∗*^				

Wasting							
Baseline	23	18.9	13	13.7	11	11	0.244
Endpoint	17	13.9	10	10.5	6	6	0.156
Change	−6	5	−3	3.2	−5	5	
*p* value^2^	0.12		0		0		

^1^McNemar's test. ^2^Chi-square test of independence. ^*∗*^Significant at *α* = 0.05.

## Data Availability

The data in this study are not available for public use because of the confidentiality agreement between the researchers and the respondents' parents as stipulated in the Ethics Protocol of Food and Nutrition Research Institute.

## Appendix

Average scores of the acceptability test using 5-point Facial Hedonic Scale (Figures 1–4).
